# Supplementary material for: Characterization of Microbial Communities from the Alimentary Canal of Typhaea stercorea (L.) (Coleoptera: Mycetophagidae)
Source: Insects. 2022 Jul 29;13(8):685. doi: 10.3390/insects13080685 (PMC9408915; doi:10.3390/insects13080685)
Supplement: Supplementary file 1 [file insects-13-00685-s001.zip › insects-1804975-supplementary.pdf]

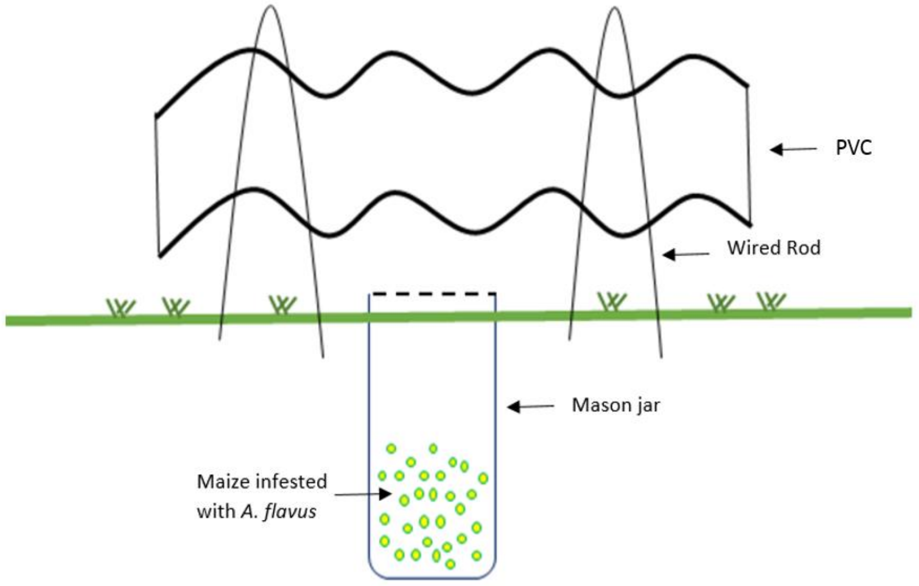

Figure S1. Diagram of the pitfall trap used to attract field strains of *T. stercorea*.

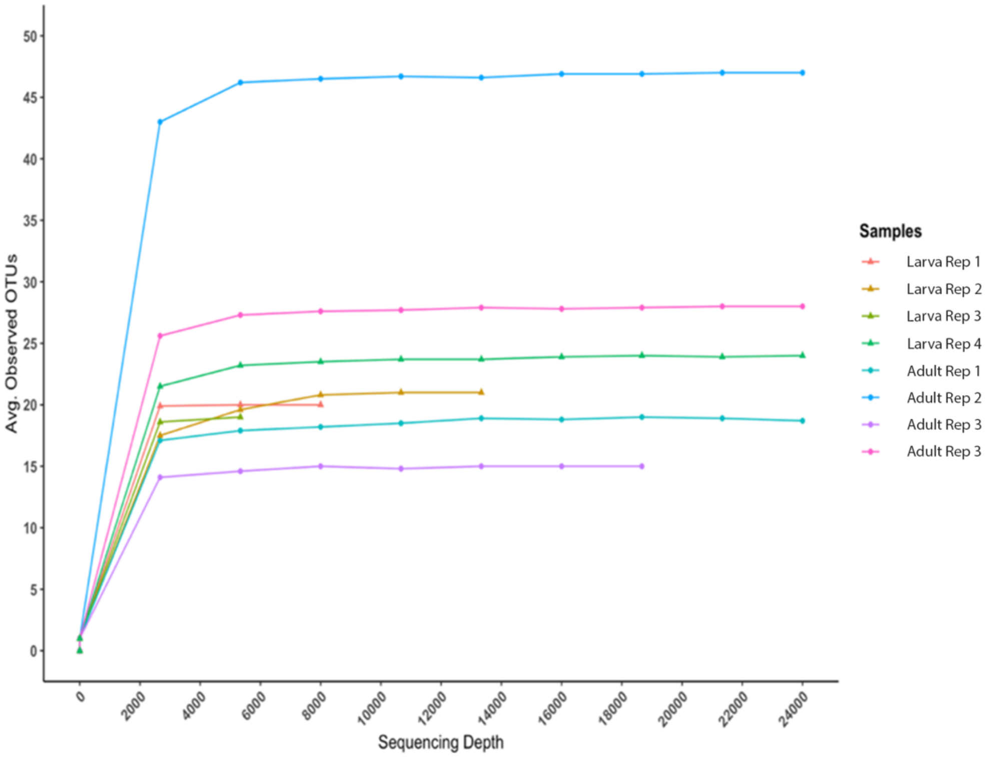

Figure S2. Alpha–rarefaction curve of the averaged observed OTUs detected in the *T. stercorea*'s alimentary canal. The sequence data were rarefied to a sequence depth of 7848 sequence count, and results are plotted as an alpha–rarefaction curve using a max depth of 24,000. Each line represents individual samples of either laboratory-reared larvae or adults. Filled triangles (▲) denote larvae and filled circles (●) denote adults.

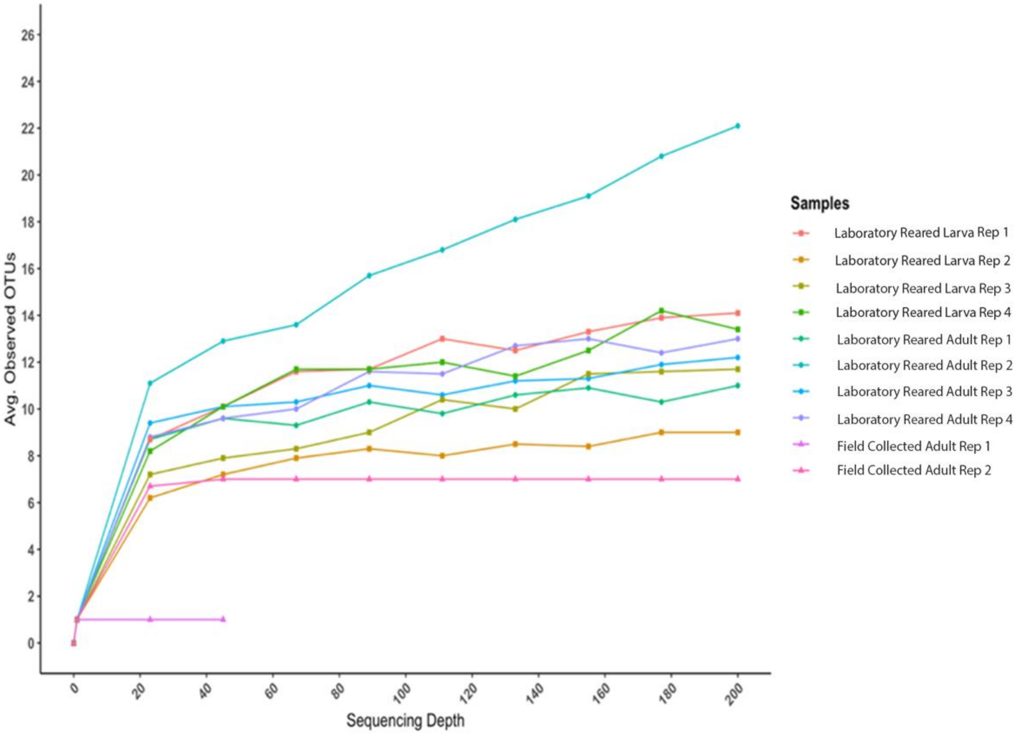

Figure S3. Alpha–rarefaction curve of the averaged observed OTUs detected in *T. stercorea*'s alimentary canal. The sequence data were rarefied to a sequence depth of 66 sequence count, and results are plotted as an alpha–rarefaction curve using a max depth of 200. Each line represents individual samples of either laboratory-reared larvae, laboratory-reared adults or field-collected adults. Filled squares (■) denote laboratory-reared larvae, filled circles (●) denote laboratory-reared adults, and filled triangles (▲) denote field-collected adults.
